# Supplementary material for: Marine-Derived Natural Product HDYL-GQQ-495 Targets P62 to Inhibit Autophagy
Source: Mar Drugs. 2023 Jan 20;21(2):68. doi: 10.3390/md21020068 (PMC9965018; doi:10.3390/md21020068)
Supplement: Supplementary file 1 [file marinedrugs-21-00068-s001.zip › marinedrugs-2159601-supplementary.pdf]

Marine-derived natural product HDYL-GQQ-495 targets P62 to inhibit autophagy

Quanfu Li [1,2,†], Jianjun Fan [3,†], Yinghan Chen [4], Yiyang Liu [1], Hang Liu [3], Wei Jiang [1,*], Dehai Li [4,*] and Yongjun Dang [3,1,*]

[1] Key Laboratory of Metabolism and Molecular Medicine, The Ministry of Education, Department of Biochemistry and Molecular Biology, School of Basic Medical Sciences, Shanghai Medical College, Fudan University, Shanghai 200032, China

[2] Department of Immunology, School of Basic Medical Sciences, Shanghai Medical College, Fudan University, Shanghai 200032, China

[3] Center for Novel Target and Therapeutic Intervention, Institute of Life Sciences, Chongqing Medical University, Chongqing 400016, China

[4] Key Laboratory of Marine Drugs, Chinese Ministry of Education, School of Medicine and Pharmacy, Ocean University of China, Qingdao 266003, China

* Correspondence: jiangw@fudan.edu.cn (W.J.); dehaili@ouc.edu.cn (D.L.); yjdang@cqmu.edu.cn (Y.D.)

† These authors contributed equally.

Supplementary Figures

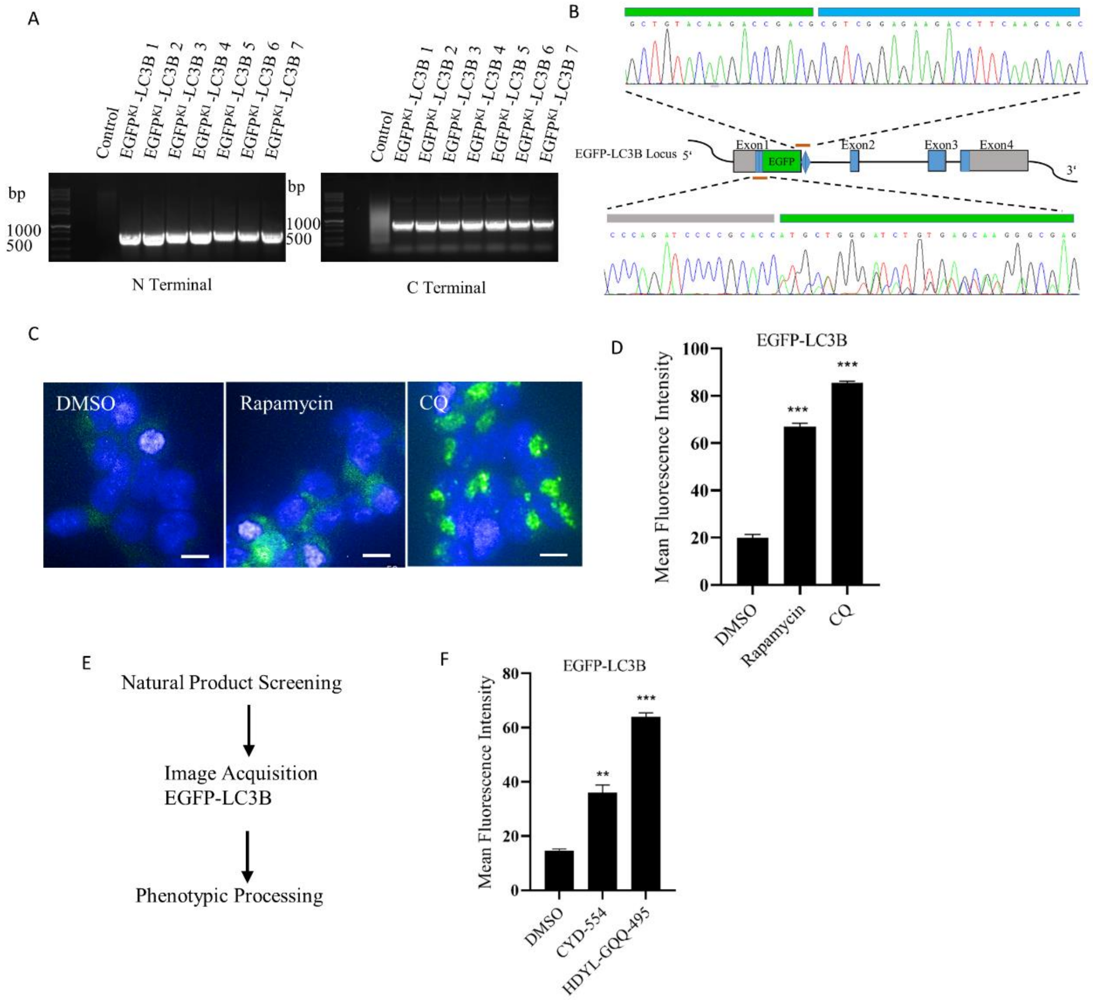

Figure S1. Identification of EGFP-LC3B knock-in 293T cell lines

(A) Genotyping PCR analysis of the integration of GFP in the indicated cells. Genomic DNA was amplified by PCR and subsequently sequenced.

(B) Representative graphic of genomic sequencing results around breakage end of the incorporated EGFP amplified from the EGFP-LC3-KI5 cell lines. The meaning of bars with different colors are listed below.

(C) Representative pictures of EGFP puncta merged with DAPI staining in the indicated cell lines with the indicated treatment. Scale bars, 10μm.

(D) Statistical analysis of the relative EGFP-LC3 expression in 293T EGFP^{KI}-LC3B with the indicated treatment. Data are shown as the mean ± SEM (n=3). ***P < 0.001, compared with DMSO.

(E) Schematic representation of the strategy of High-throughput screening in 293T EGFP^{KI}-LC3B 5 cells.

(F) Statistical analysis of the relative EGFP-LC3 expression in 293T EGFP[KI]-LC3B with the indicated treatment. Data are shown as the mean ± SEM (n=3). **P < 0.01***P < 0.001, compared with DMSO.

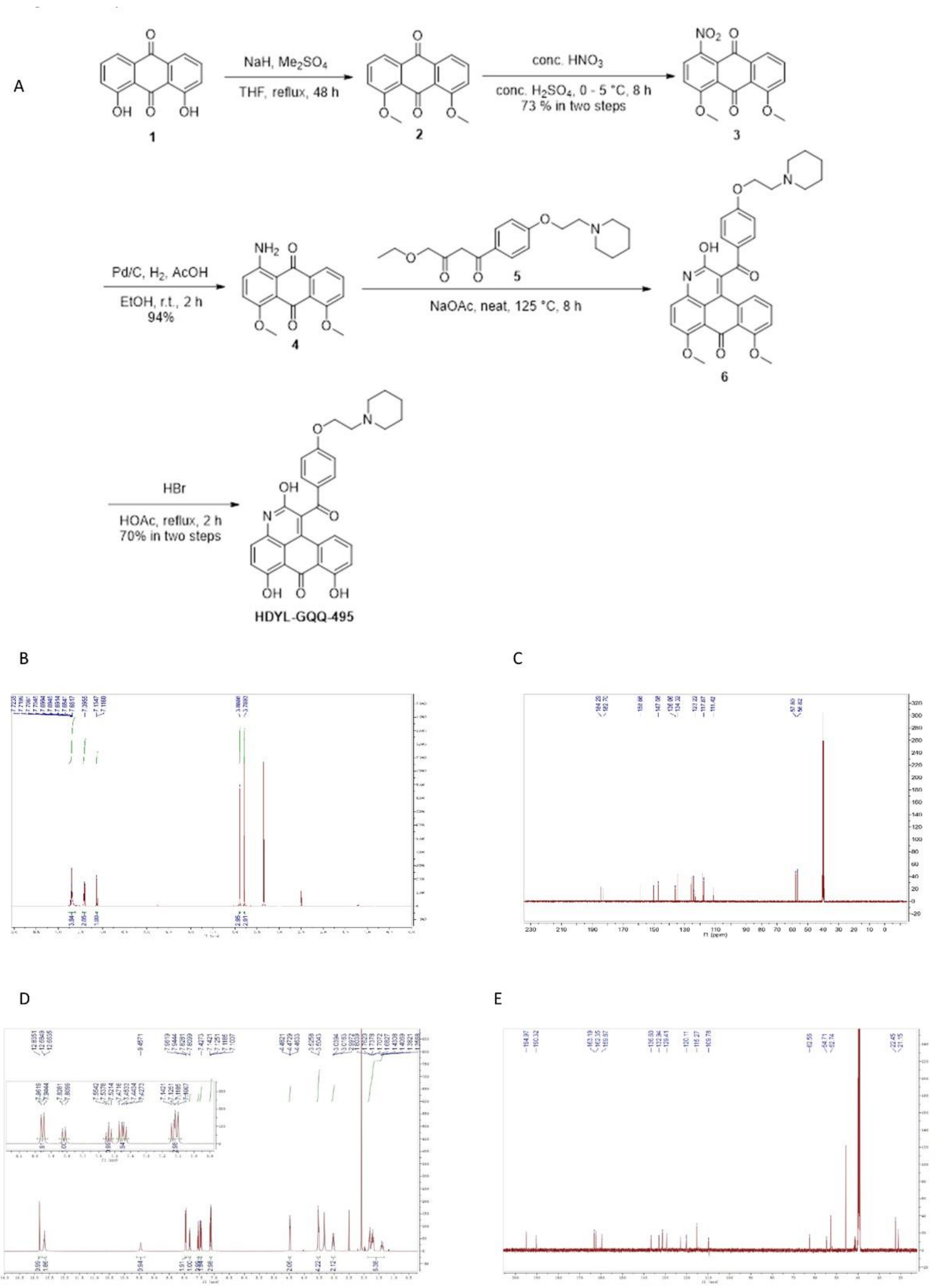

Figure S2. Synthesis HDYL-GQQ-495

(A) Designed procedure to synthesize HDYL-GQQ-495.

(B) ^{1}H NMR of **4**, d_6-DMSO.

(C) ^{13}C NMR of **4**, d_6-DMSO.

(D) ^{1}H NMR of **495**, d_6-DMSO.

(E) ^{13}C NMR of **495**, d_6-DMSO.

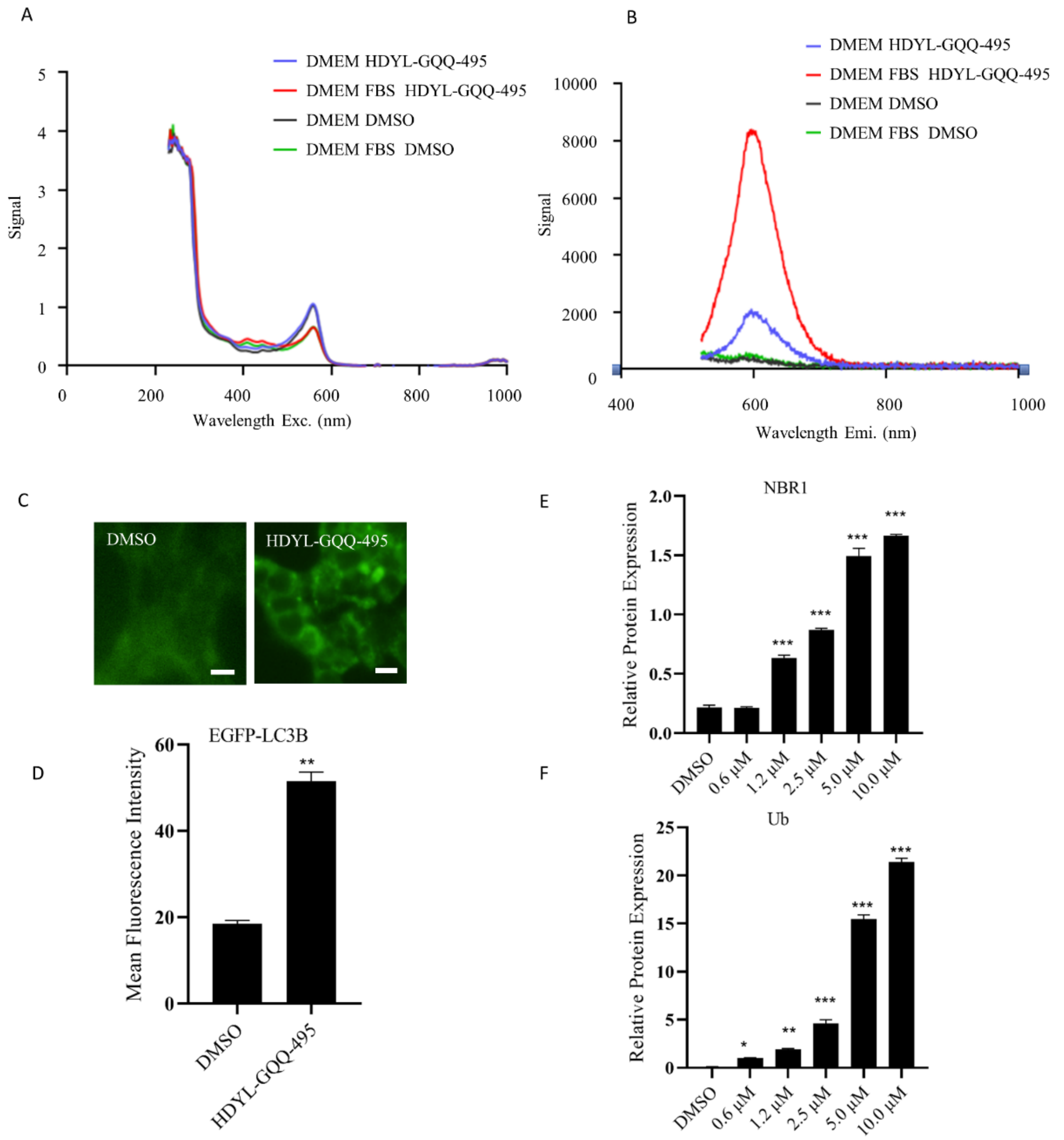

Figure S3. HDYL-GQQ-495 is a fluorescent reagent with excitation at 500 nm and emission at 590 nm

(A-B) The emission and excitation spectra spectra of GQQ-495 scanned by EnSpire.

(C) Representative pictures of EGFP puncta in the 293T EGFP^{KI}-LC3B cells with HDYL-GQQ-495 and chloroquine. Scale bars, 10μm.

(D) Statistical analysis of the relative EGFP-LC3 expression in 293T EGFP^{KI}-LC3B with the indicated treatment. Data are shown as the mean ± SEM (n=3). **P < 0.01, compared with DMSO.

(E) Statistical analysis of the relative NBR1 expression in HeLa cells with the indicated treatment. Data are shown as the mean ± SEM (n=3). ***P < 0.001, compared with DMSO.

(F) Statistical analysis of the relative Ub expression in HeLa cells with the indicated treatment. Data are shown as the mean ± SEM (n=3). *P < 0.05, **P < 0.01, ***P < 0.001, compared with DMSO.

Supplementary Table S1. Relative mean GFP fluorescence

Compounds	Relative Mean GFP
DMSO	100.00
CYD-019	81.23
CYD-008	109.21
CYD-003	96.16
CYD-049	79.94
CYD-050	80.98
CYD-061	94.37
CYD-007	112.37
CYD-018	102.46
CYD-109	111.27
CYD-101	105.45
CYD-111 （HDYL-GQQ-495）	143.36
CYD-112	86.90
CYD-113	80.45
CYD-114	118.88
CYD-115	109.90
CYD-116	115.56
CYD-117	96.59
CYD-118	86.52
CYD-119	88.88
CYD-220	87.93
CYD-221	99.56
CYD-222	102.34
CYD-223	89.94
CYD-224	105.78
CYD-225	80.90
CYD-226	118.88
CYD-227	83.45
CYD-228	89.93
CYD-229	92.40
CYD-230	95.69
CYD-331	90.80
CYD-332	100.34
CYD-333	118.98
CYD-334	105.24

CYD-335	109.98
CYD-336	88.79
CYD-337	80.80
CYD-338	83.59
CYD-339	86.39
CYD-440	98.84
CYD-441	104.87
CYD-442	110.34
CYD-443	119.43
CYD-444	89.99
CYD-445	80.34
CYD-446	90.36
CYD-447	83.24
CYD-448	97.24
CYD-449	92.46
CYD-450	100.35
CYD-451	118.40
CYD-452	83.33
CYD-553	136.45
CYD-554	102.93
CYD-555	114.34
CYD-556	108.46
CYD-557	89.42
CYD-558	93.35
CYD-559	112.39
CYD-560	94.89
CYD-161	86.39
CYD-262	90.38
CYD-263	108.35
CYD-264	119.44
CYD-265	87.77
CYD-246	83.59
CYD-267	109.24
CYD-268	113.24
CYD-569	112.49
CYD-270	100.03
CYD-371	89.94
CYD-022	84.30
CYD-173	94.49

CYD-074	99.98
CYD-275	108.09
CYD-076	117.77
CYD-177	109.56
CYD-554	129.50
CYD-345	116.38
CYD-137	119.34
CYD-121	108.79
CYD-502	99.38
H-002	90.78
H-003	94.38
H-004	106.89
H-005	110.38
H-006	117.39
H-007	113.21
H-008	115.89
H-009	116.47
H-010	102.53
H-011	87.95
H-012	117.74
H-013	117.51
H-014	109.09
H-015	91.34
H-016	92.16
H-017	94.41
H-018	90.00
H-019	103.16
H-020	97.99
H-021	115.54
H-022	117.43
H-023	107.04
H-024	89.54
H-025	85.99
H-026	90.47
H-027	91.83
H-028	91.68
H-029	113.26
H-030	111.04
H-031	90.70

H-032	113.93
H-033	112.82
H-034	85.42
H-035	88.66
H-036	113.67
H-037	130.48
H-038	96.36
H-039	93.28
H-040	83.15
H-041	110.90
H-042	88.60
H-043	98.99
H-044	91.94
H-045	85.55
H-046	90.45
H-047	106.86
H-048	105.78
H-049	113.71
H-050	115.72
H-051	109.65
H-052	82.00
H-053	98.22
H-054	95.45
H-055	116.60
H-056	107.40
H-057	113.79
H-058	114.00
H-059	107.62
H-060	88.22
H-061	108.68
H-062	93.83
H-063	105.42
H-064	88.77
H-065	106.45
B-103	96.99
B-104	117.45
B-105	98.20
B-106	108.83
B-107	103.60

B-108	92.53
B-109	101.17
B-110	91.52
B-111	103.18
B-112	93.30
B-113	89.47
B-114	109.36
B-115	100.08
B-116	90.43
B-117	107.42
B-118	82.90
B-119	132.23
B-120	102.83
B-121	116.82
B-122	118.70
B-123	91.09
B-124	119.14
B-125	94.64
B-126	106.84
B-127	100.32
B-128	108.96
B-129	115.95
B-130	108.88
B-131	102.37
B-132	115.55
B-133	105.42
B-134	105.30
B-135	100.65
B-136	107.97
B-137	116.60
B-138	105.47
B-139	101.73
B-140	106.23
B-141	96.78
B-142	112.80
B-143	82.71
B-144	83.60
B-145	88.78
B-146	103.16

B-147	100.34
B-148	113.88
B-149	106.66
B-150	106.05
B-151	110.37
B-152	88.41
B-153	119.71
B-154	113.86
B-155	115.81
B-156	96.61
I-100	93.37
I-101	95.63
I-102	97.60
I-103	98.28
I-104	91.30
I-105	94.96
I-106	83.32
I-107	103.14
I-108	102.34
I-109	106.13
I-110	96.25
I-111	105.08
I-112	81.97
I-113	126.40
I-114	99.53
I-115	105.03
I-116	92.10
I-117	119.52
I-118	108.37
I-119	107.24
I-120	101.38
I-121	119.57
I-122	105.77
I-123	91.69
I-124	84.08
I-125	98.82
I-126	88.03
I-127	95.98
I-128	119.73

I-129	81.64
I-130	107.43
I-131	90.40
I-132	103.88
I-133	102.92
I-134	100.35
I-135	107.75
I-136	116.02
I-137	116.30
I-138	107.34
I-139	84.38
I-140	114.17
I-141	111.76
I-142	93.99
I-143	113.20
I-144	119.61
I-145	85.57
I-146	118.18
I-147	112.43
G-002	88.19
G-003	108.90
G-004	84.44
G-005	84.84
G-006	105.81
G-007	101.29
G-008	81.55
G-009	86.63
G-010	87.51
G-011	124.35
G-012	106.90
G-013	102.08
G-014	89.21
G-015	106.38
G-016	84.42
G-017	90.32
G-018	85.98
G-019	81.00
G-020	82.01
G-021	119.96

G-022	110.98
G-023	84.83
G-024	91.42
G-025	107.91
G-026	83.52
G-027	88.17
G-028	91.14
G-029	114.05
G-030	107.83
G-031	111.52
G-032	113.97
G-033	92.00
G-034	106.67
G-035	83.94
G-036	99.69
G-037	83.78
G-038	111.99
G-039	107.14
G-040	87.76
G-041	93.72
G-042	92.03
G-043	81.68
G-044	113.96
G-045	101.17
G-046	110.99
G-047	118.53
G-048	81.64
G-049	112.78
G-050	90.13
G-051	110.09
G-052	84.75
G-053	84.14
G-054	91.45
G-055	107.41
G-056	116.24
G-057	80.02
G-058	103.12
G-059	119.47
G-060	97.63

G-061	115.67
G-062	114.53
G-063	83.63
G-064	109.17
G-065	91.25
G-066	104.82
P-11	100.66
P-12	101.19
P-13	107.18
P-14	96.26
P-15	116.11
P-16	112.17
P-17	118.37
P-18	98.10
P-19	80.12
P-20	80.01
P-21	108.55
P-22	119.02
P-23	111.27
P-24	93.92
P-25	99.62
P-26	108.19
P-27	84.29
P-28	98.69
P-29	80.62
P-30	117.39
P-31	92.31
P-32	111.64
P-33	100.38
P-34	100.46
P-35	114.61
P-36	106.89
P-37	118.78
P-38	104.17
P-39	95.30
P-40	97.53
P-41	106.76
P-42	101.54
P-43	86.70

P-44	92.22
P-45	120.73
P-46	106.08
P-47	112.74
K-015	87.02
K-016	99.62
K-017	91.55
K-018	82.35
K-019	85.21
K-020	91.96
K-021	80.83
K-022	88.85
K-023	86.40
K-024	101.23
K-025	93.07
K-026	99.09
K-027	80.42
K-028	83.02
K-029	94.37
K-030	109.49
K-031	88.81
K-032	110.23
K-033	113.09
K-034	90.84
K-035	106.10
K-036	116.18
K-037	86.72
K-038	85.48
K-039	107.15
K-040	94.70
K-041	83.41
K-042	102.91
K-043	119.77
K-044	93.02
K-045	92.11
K-046	97.52
K-047	82.64
K-048	99.80
K-049	103.86

K-050	119.59
K-051	105.49
K-052	118.04
K-053	94.88
K-054	106.33
K-055	82.77
K-056	96.69
K-057	93.52
K-058	119.54
K-059	111.13
K-060	83.33
K-061	94.57
K-062	111.31
K-063	102.54
K-064	95.88
K-065	109.71
K-066	109.92
K-067	116.68
K-068	96.97
K-069	102.22
K-070	110.58
K-071	91.59
K-072	86.93
K-073	101.82
M-12	108.88
M-13	103.08
M-14	87.66
M-15	106.49
M-16	102.80
M-17	98.67
M-18	88.41
M-19	80.71
M-20	90.28
M-21	91.21
M-22	84.37
M-23	118.55
M-24	91.46
M-25	108.85
M-26	104.21

M-27	84.84
M-28	97.81
M-29	95.94
M-30	84.65
M-31	111.05
M-32	81.26
M-33	84.87
M-34	108.71
M-35	107.97
M-36	88.36
M-37	94.72
M-38	80.03
M-39	114.95
M-40	95.74
M-41	116.32
M-42	102.70
M-43	95.59
M-44	102.15
M-45	96.47
M-46	101.09
M-47	113.10
M-48	91.08
M-49	101.50
M-50	92.12
M-51	83.83
M-52	108.95
M-53	114.75
M-54	108.88
M-55	115.01
M-56	114.13
M-57	91.10
M-58	112.20
M-59	106.52
M-60	100.56
M-61	91.76
M-62	104.85
M-63	82.10
M-64	111.35
M-65	97.98

M-66	85.97
M-67	105.54
M-68	118.86
M-69	109.72
M-70	106.92
M-71	88.70
M-72	92.58
M-73	103.31
M-74	83.48
M-75	86.24
M-76	97.84
M-77	107.43
M-78	100.78
M-79	110.58
M-80	84.95
M-81	101.02
M-82	110.59
M-83	84.83
M-84	84.37
M-85	110.98
M-86	84.77
M-87	96.40
M-88	112.19
M-89	87.13
M-90	87.93
M-91	88.23
M-92	90.90
